# Genome size variation and incidence of polyploidy in Scrophulariaceae *sensu lato* from the Iberian Peninsula

**DOI:** 10.1093/aobpla/pls037

**Published:** 2012-11-28

**Authors:** Mariana Castro, Sílvia Castro, João Loureiro

**Affiliations:** CFE, Centre for Functional Ecology, Department of Life Sciences, University of Coimbra, PO Box 3046, Coimbra 3001-401, Portugal

## Abstract

**Background and aims:**

In the last decade, genomic studies using DNA markers have strongly influenced the current phylogeny of angiosperms. Genome size and ploidy level have contributed to this discussion, being considered important characters in biosystematics, ecology and population biology. Despite the recent increase in studies related to genome size evolution and polyploidy incidence, only a few are available for Scrophulariaceae. In this context, we assessed the value of genome size, mostly as a taxonomic marker, and the role of polyploidy as a process of genesis and maintenance of plant diversity in Scrophulariaceae *sensu lato* in the Iberian Peninsula.

**Methodology:**

Large-scale analyses of genome size and ploidy-level variation across the Iberian Peninsula were performed using flow cytometry. One hundred and sixty-two populations of 59 distinct taxa were analysed. A bibliographic review on chromosome counts was also performed.

**Principal results:**

From the 59 sampled taxa, 51 represent first estimates of genome size. The majority of the Scrophulariaceae species presented very small to small genome sizes (2C ≤ 7.0 pg). Furthermore, in most of the analysed genera it was possible to use this character to separate several taxa, independently if these genera were homoploid or heteroploid. Also, some genome-related phenomena were detected, such as intraspecific variation of genome size in some genera and the possible occurrence of dysploidy in *Verbascum* spp. With respect to polyploidy, despite a few new DNA ploidy levels having been detected in *Veronica*, no multiple cytotypes have been found in any taxa.

**Conclusions:**

This work contributed with important basic scientific knowledge on genome size and polyploid incidence in the Scrophulariaceae, providing important background information for subsequent studies, with several perspectives for future studies being opened.

## Abstract (precis)

In this work, genome size variation and polyploidy incidence in Scrophulariaceae from the Iberian Peninsula were addressed, with the obtained results providing important background information for subsequent studies, with several future perspectives being opened.

## Introduction

Knowledge of the genome has been increasingly important in many areas of plant research, including taxonomy and biosystematics, ecology and population biology. Genomes represent a distinct and legitimate level of organization, with unique and particular evolutionary histories. Genome size is one of its intrinsic characteristics, being considered a constant species-specific character that can help to explain relationships between species (Gregory 2001). As shown by Bennett and Leitch (2010), genome size is still unknown for ∼97.5 % of angiosperm species. Despite the small representation of estimates it is already possible to find a large variation in genome size among different taxonomic groups. This highlights the relevance of genome size as a taxonomic and/or ecological marker in particular plant groups. Also, the variation in the amount of DNA content (or lack of it) has been a central focus of evolutionary biology, and an important tool to know the structure of genetic information, its evolution and function, and to understand the biological basis of the diversity and its adaptive value in ecological, evolutionary and taxonomic interpretations (Gregory 2005*b*; Greilhuber *et al.* 2010).

Nowadays, genomes are considered to be highly dynamic and their evolution is considered to be a bidirectional process, with its size resulting from a dynamic balance between expansion and contraction forces (Bennett and Leitch 2005). Generally, polyploidy is one of the mechanisms that may lead to increases in genome size. In homoploid plants (i.e. species with the same number of chromosomes), genome expansion is due to amplification and insertion of transposable genetic elements (different amounts of non-coding, repetitive DNA sequences; Vitte and Bennetzen 2006) and evolution and amplification of satellite repeats (variation in the number and proportion of minisatellites and microsatellites; Lim *et al*. 2006). Relative to the loss of genome size, it is associated with deletional mechanisms such as unequal intra-strand homologous recombination, illegitimate recombination and/or higher rate of nucleotide deletion over insertion (Bennetzen *et al*. 2005).

In biosystematics, ecology and evolution, genome size has been important as a tool to discriminate taxa and resolve complex low-level taxonomies, to distinguish groups with phenotypic similarities, with a low number of distinct morphological characters, with continuous morphological variations and/or groups prone to inter-specific hybridization or with complex evolutionary histories (e.g. allopolyploids). Also, several studies tried to predict the correlation of genomes size with several phenotypic, physiological and/or ecological characteristics (the nucleotypic effect) and to understand the dynamics of genome evolution (studying inter- and intraspecific variation ‘patterns’ in genome size) (Loureiro *et al.* 2010).

As traditionally circumscribed (e.g. von Wettstein 1891), the Scrophulariaceae is the largest family within the order Lamiales and has a worldwide distribution. However, recent molecular studies using DNA sequences of plastid genes revealed at least five distinct monophyletic groups, leading to the disintegration of the traditional classification of Scrophulariaceae *sensu lato* (*s.l.*) in, at least, six families (Olmstead *et al*. 2001). Members of the classical Scrophulariaceae are currently found in Scropulariaceae *sensu stricto* (*s.s.*), Plantaginaceae, Orobanchaceae (the latter two contain most of the taxa that have moved), Stilbaceae, Phrymaceae and Linderniaceae (Olmstead *et al*. 2001; The Angiosperm Phylogeny Group 2003). In the Iberian Peninsula, Scrophulariaceae *s.l*. is represented by 323 species distributed in 33 genera (Benedí *et al.* 2009). Most species are ruderal and can be easily found in disturbed lands; however, there are several species listed in the red lists, and thus in need of special protection (e.g. *Anarrhinum longipedicellatum*).

Considering that there is almost no available information on genome size for any taxa of this family (but see Albach and Greilhuber 2004), that there are several records in the literature pointing to the possible existence of polyploids within and between species of Scrophulariaceae (e.g. *Antirrhinum*, *Digitalis* and *Veronica*) and that, in case polyploids are found, many taxa present large attractive flowers, ideal for reproductive isolation studies, it would be important to obtain background information on genome evolution and polyploid incidence in the Scrophulariaceae through a large-scale cytogenetic-based study.

Therefore, the main objectives of the present study were to assess the value of genome size as a taxonomic marker, and the role of polyploidy as a process of genesis and maintenance of plant diversity in Scrophulariaceae *s.l.* in the Iberian Peninsula. For that we: (i) assessed chromosome numbers, genome size and polyploidy incidence in Scrophulariaceae taxa from the Iberian Peninsula through an exhaustive review of the bibliographic literature; (ii) estimated the genome size of a diverse array of taxa from several key genera; and (iii) assessed cytotype diversity through large-scale screenings in natural populations.

## Methods

### Plant material

Plant samples from 59 taxa of the Scrophulariaceae *s.l.* were collected from several field locations in Portugal and Spain. Seeds from some taxa were kindly provided by *Index Semina* of several Iberian research institutions [see Additional Information].

Field collections were carried out during the flowering season (March to August) of the studied taxa. In each population, leaves and/or seeds from up to 30 individuals were collected and stored in hermetic plastic bags. Samples were kept at 4 °C in a refrigerator until analysis (usually, not >2 days). Voucher specimens were also collected for plant identification and were kept in the Herbarium of the University of Coimbra.

Seeds from Scrophulariaceae taxa and from reference standards were sown in plastic cuvettes filled with commercial peat. Plastic cuvettes were put in a greenhouse operating at 20 ± 2 °C and with a photoperiod of 16 h/8 h (light/dark) and a light intensity of 530 ± 2 µmol m^−2^ s^−1^.

### Bibliographic review

An extensive bibliographic review on chromosome counts, localities and genome size of the studied species was carried out. For chromosome information and localities the following bibliography or online databases were used: Flora Iberica (Benedí *et al.* 2009), Tropicos® (Website 1), Anthos (Aedo and Castroviejo, 2005), BioDiversity4all (Ribeiro *et al.* 2011; for localities only) and M. Queirós printed files database available at the Department of Life Sciences, University of Coimbra. For genome size information, the Plant DNA C-values Database (Bennett and Leitch 2010) was the main source of information.

### Genome size and ploidy-level estimations

Flow cytometric (FCM) analyses of genome size and ploidy level were carried out using leaves from field-collected or seed-germinated plants. Nuclear suspensions were prepared according to Galbraith *et al*. (1983), by chopping ∼50 mg of plant material of the sample species and ∼50 mg of leaves of the internal reference standard (when possible and justifiable, the same reference standard was used for all the taxa of each genus and prior to this study their genome size was recalibrated using *Pisum sativum* ‘Ctirad’ as the primary standard [see Additional Information]) with a sharp razor blade in a glass Petri dish containing 1 mL of WPB buffer (0.2 M Tris-HCl, 4 mM MgCl_2_·6H_2_O, 1 % Triton X-100, 2 mM EDTA Na_2_ 2H_2_O, 86 mM NaCl, 10 mM metabisulfite, 1 % PVP-10, pH adjusted to 7.5 and stored at 4 °C; Loureiro *et al*. 2007*a*). For each taxon/population, after the first sample, if necessary the chopping intensity and amount of plant material were adjusted in order to have a rate of 20–50 nuclei/s in subsequent replicates. In samples with a large amount of cytosolic compounds, the chopping intensity was reduced to avoid their release from the cells and thus prevent or minimize their negative effect on nuclear fluorescence (Loureiro *et al*. 2006). Nuclear suspensions were then filtered through a 50-µm nylon filter and 50 µg mL^−1^ propidium iodide (PI; Fluka, Buchs, Switzerland) and 50 µg mL^−1^ RNAse (Fluka) were added to sample tubes to stain the DNA and avoid staining of double-stranded RNA, respectively. Samples were analysed within a 5-min period in a Partec CyFlow Space flow cytometer (Partec GmbH, Görlitz, Germany) equipped with a 532 nm green solid-state laser, operating at 30 mW. Integral fluorescence and fluorescence height and width emitted from nuclei were collected through a 620-nm band-pass interference filter. For each taxon, the amplifier system was set to a constant voltage and gain. Each day, prior to analysis, the instrument stability and linearity were checked either with fluorescent beads or using PI-stained nuclei isolated from *P. sativum* ‘Ctirad’. The analyses were only started when CV values were <2 %. If this was not achieved both a cleaning procedure and an adjustment of the position of the flow chamber with respect to the incident laser were made until the optimal CV values were obtained.

Results were acquired using the Partec FloMax software (v. 2.5) in the form of six graphics: fluorescence pulse integral in linear scale (FL); forward light scatter (FS) vs. side light scatter (SS), both in logarithmic (log) scale; FL vs. time; FL vs. fluorescence pulse height; FL vs. FS in log scale and FL vs. SS in log scale (for an example of data acquisition see [Additional Information]). In most samples, in the latter graphic, a polygonal region was defined to include only intact nuclei, which was subsequently used to gate all the other graphics. At least 1300 nuclei in both sample and standard G_1_ peaks were analysed per sample (Suda *et al*. 2007). Genome size estimates were only considered when the CV values of G_1_ peaks were <5 %. Samples with higher CV values were discarded and a new sample was prepared. For some taxa with high amounts of cytosolic compounds it was not possible to achieve such CV values, and thus a higher CV threshold was considered acceptable (8 %).

In each population, ploidy level and genome size were obtained for three individuals. For the remaining individuals, only ploidy-level information was gathered. For those individuals, the pooled sample strategy (5–6 individuals plus the reference standard) was followed.

Ploidy-level analyses consisted of determining the DNA index (ratio between the mean FL of sample and standard *G*_1_ nuclei), with the assumed DNA ploidy level being in most cases the one more commonly found in the literature (Suda *et al.* 2007). The holoploid genome size in pg (2C; *sensu* Greilhuber *et al*. 2005) of each individual was estimated by multiplying the DNA index by the nuclear DNA content of the reference standard. The monoploid genome size (1Cx; *sensu* Greilhuber *et al*. 2005) of all species was also calculated by dividing the holoploid genome size (2C) by the supposed ploidy level of each taxa in mass values (pg).

### Statistical analyses

Descriptive statistics of genome size were calculated for each taxon (mean, standard deviation of the mean and coefficient of variation of the mean). For genera with more than one species, box plots with mean and standard deviation of the mean were computed.

Differences in genome size among families considering the newly established circumscriptions (i.e. Scrophulariaceae *s.s*., Orobanchaceae and Plantaginaceae) were assessed using a non-parametric Kruskal–Wallis one-way analysis of variance (ANOVA) on ranks (normal distribution data and homoscedasticity was not achieved even after data transformation). For genera with more than one sampled species (*Anarrhinum*, *Antirrhinum*, *Digitalis*, *Linaria*, *Misopates*, *Pedicularis*, *Scrophularia*, *Verbascum* and *Veronica*) differences in nuclear DNA content within and between species were evaluated. For variables that were normally distributed and homoscedastic, a *t-*test or a one-way ANOVA was followed. In *Linaria* spp. and *Veronica* spp., data transformations (log_10_ and square root, respectively) had to be used to achieve normal distribution data and homoscedasticity. In *Scrophularia* sp., due to failure in achieving homoscedasticity, even after data transformation, a non-parametric Kruskal–Wallis one-way ANOVA on ranks was used. When statistically significant differences were detected, either a multiple comparison Tukey–Kramer test (for parametric data) or Dunn's method (for non-parametric data) was applied to determine which groups presented significantly different values. In *Veronica* spp., a linear regression analysis and a Pearson correlation were performed between mean nuclear DNA content and chromosome numbers of each taxon. All statistical analyses were carried out using SPSS software (IBM Corporation, Somers, NY, USA).

## Results

The bibliographic review on chromosome counts of 116 Scrophulariaceae *s.l.* taxa present in the Iberian Peninsula revealed that 28 taxa presented more than one value of chromosome numbers, despite that only in 10 taxa (8.6 % of the total) this may represent different ploidy levels (e.g. *Digitalis purpurea* subsp. *purpurea* and *Odontites vernus*, both with 2*x* and 4*x*; *Veronica cymbalaria* and *V. hederifolia*, both with 2*x* and 3*x*). For the remaining taxa, usually differences of two or more chromosomes are reported, but never an additional full set of chromosomes [see Additional Information].

**Fig. 1:**
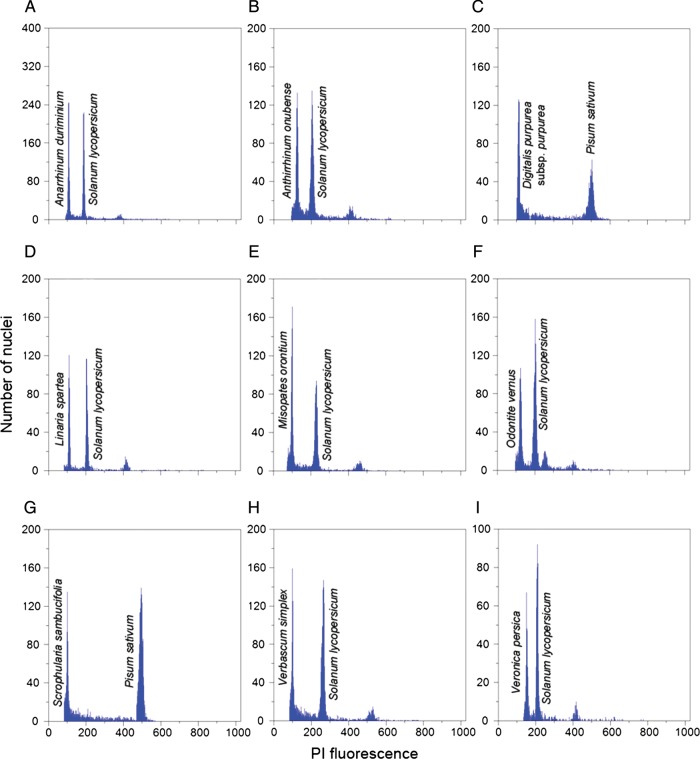
**Flow cytometric histograms of relative PI fluorescence intensity obtained after simultaneous analysis of nuclei isolated from the internal reference standard and from the Scrophulariaceae species.** (A) *G*_1_ peaks of *Anarrhinum duriminium* and *Solanum lycopersicum*; (B) *G*_1_ peaks of *Antirrhinum onubense* and *S. lycopersicum*; (C) *G*_1_ peaks of *Digitalis purpurea* subsp. *purpurea* and *Pisum sativum*; (D) *G*_1_ peaks of *Linaria spartea* and *S. lycopersicum*; (E) *G*_1_ peaks of *Misopates orontium* and *S. lycopersicum*; (F) *G*_1_ peaks of *Odontite vernus* and *S. lycopersicum*; (G) *G*_1_ peaks of *Scrophularia sambucifolia* and *P. sativum*; (H) *G*_1_ peaks of *Verbascum simplex* and *S. lycopersicum*; (I) *G*_1_ peaks of *Veronica persica* and *S. lycopersicum*. In histograms (A), (B), (D)–(F), (H) and (I) it is possible to observe the *G*_2_ peak of the internal reference standard; additionally, in (F) it is also possible to observe the *G*_2_ peak of *O. vernus* (third peak). Also, please note the overall good quality of the histograms, as defined by the narrow *G*_1_ peaks and by the low amount of background debris.

The use of FCM enabled us to perform a large-scale analysis of 17 genera of Scrophulariaceae *s.l.*, comprising 59 species and a total of 162 populations (Table 1). From the 59 sampled species, 51 are first estimations of genome size (86 %, Table 1). With a few exceptions (e.g. *Veronica micrantha*) the overall quality of the results, as given by the CV values of G_1_ peaks and by the background debris, was good, with mean CV values <5 % being achieved in most taxa (Fig. 1).

**Table 1:** Nuclear DNA content estimations in the studied taxa of Scrophulariaceae *s.l.* The values are given as mean and standard deviation of the mean of the holoploid genome size (2C, pg) of individuals of each species. For each species, the monoploid genome size in mass values (1Cx, pg), the mean coefficient of variation (CV, %) of *G*_0_/*G*_1_ peaks, the supposed ploidy level (^2^), the reference standard used to estimate the genome size (R.s.^3^), the number of individuals analysed for genome size (*n* G.s.), the total number of analysed individuals (*n* total), the total number of analysed populations (*n* Pop.) and the origin of plant material (POP, natural populations; IS, *index seminum*) are also given. Also, for each species, previous genome size estimations and original references are provided (^A^Mowforth 1986; ^B^Nagl and Fusenig 1979; ^C^Albach and Greilhuber 2004; ^D^Bennett 1972). In bold are highlighted the new DNA ploidy levels that were assumed in this study. ^1^1 pg = 978 Mbp (Doležel *et al.* 2003). ^3^R, *Raphanus sativus* ‘Saxa’; S, *Solanum lycopersicum* ‘Stupické’; G, *Glycine max* ‘Polanka’; B, *Bellis perenis*; Z, *Zea mays* ‘CE-777’; P, *Pisum sativum* ‘Ctirad’.

Taxon	Family *s.s.*	Genome size (2C, pg)		Genome size (1Cx, pg^1^)	FL CV (%)	Ploidy level^2^	R.s.^3^	*n* G.s.	*n* total	*n* Pop.	Origin	Previous estimations
		Mean ± SD	CV (%)									
*Anarrhinum bellidifolium*	Plantaginaceae	1.13 ± 0.03	3.1	0.56	3.68	2*n* = 2*x*	S	32	157	11	POP + IS	First estimation
*Anarrhinum duriminium*	Plantaginaceae	1.11 ± 0.02	2.2	0.56	5.61	2*n* = 2*x*	S	12	35	3	POP + IS	First estimation
*Anarrhinum longipedicelatum*	Plantaginaceae	1.12 ± 0.02	1.4	0.57	4.05	2*n* = 2*x*	S	7	40	3	POP	First estimation
*Antirrhinum cirrhigerum*	Plantaginaceae	1.21 ± 0.01	0.8	0.59	5.95	2*n* = 2*x*	S	3	30	1	IS	First estimation
*Antirrhinum graniticum*	Plantaginaceae	1.18 ± 0.05	3.9	0.59	5.06	2*n* = 2*x*	S	3	30	1	IS	First estimation
*Antirrhinum linkianum*	Plantaginaceae	1.23 ± 0.03	2.7	0.61	4.66	2*n* = 2*x*	S	17	66	7	POP + IS	First estimation
*Antirrhinum meonanthum*	Plantaginaceae	1.20	–	0.61	5.36	2*n* = 2*x*	S	1	1	1	IS	First estimation
*Antirrhinum onubense*	Plantaginaceae	1.18 ± 0.01	1.1	0.61	4.15	2*n* = 2*x*	S	3	20	1	POP	First estimation
*Bartsia trixago*	Orobanchaceae	1.85 ± 0.08	4.1	0.93	3.91	2*n* = 2*x*	G/S	17	103	6	POP + IS	First estimation
*Chaenorhinum origanifolium*	Plantaginaceae	1.13 ± 0.02	1.3	0.57	3.47	2*n* = 2*x*	S	9	61	3	POP	First estimation
*Cymbalaria muralis* subsp. *muralis*	Plantaginaceae	0.99 ± 0.02	2.5	0.49	5.12	2*n* = 2*x*	S	9	48	3	POP	First estimation
*Digitalis mariana* subsp. *heywoodii*	Plantaginaceae	1.12	–	0.56	6.77	2*n* = 2*x*	S	1	1	1	IS	First estimation
*Digitalis purpurea* subsp*. purpurea*	Plantaginaceae	1.87 ± 0.05	2.6	0.94	3.64	2*n* = 2*x*	B/P	19	168	11	POP + IS	2C = 2.45 pg^A^
*Digitalis thapsi*	Plantaginaceae	2.08	–	1.04	5.90	2*n* = 2*x*	Z	1	1	1	IS	First estimation
*Euphrasia minimus*	Orobanchaceae	1.29 ± 0.02	1.3	0.65	3.02	2*n* = 2*x*	S	3	30	1	POP	First estimation
*Kickxia spuria* subsp. *integrifolia*	Plantaginaceae	1.64 ± 0.02	1.2	0.82	3.44	2*n* = 2*x*	S	4	17	1	IS	First estimation
*Linaria aeruginea* subsp*. aeruginea*	Plantaginaceae	1.29 ± 0.01	0.9	0.64	3.83	2*n* = 2*x*	S	2	5	1	POP	First estimation
*Linaria amethystea* subsp. *amethystea*	Plantaginaceae	1.05 ± 0.01	0.6	0.53	3.67	2*n* = 2*x*	S	3	30	1	POP	First estimation
*Linaria diffusa*	Plantaginaceae	1.15 ± 0.00	0.4	0.57	2.97	2*n* = 2*x*	S	2	15	1	POP	First estimation
*Linaria incarnata*	Plantaginaceae	1.13 ± 0.00	0.3	0.56	3.31	2*n* = 2*x*	S	2	15	1	POP	First estimation
*Linaria polygalifolia* subsp*. polygalifolia*	Plantaginaceae	1.32 ± 0.04	2.7	0.66	4.17	2*n* = 2*x*	S	12	70	4	POP	First estimation
*Linaria saxatilis*	Plantaginaceae	1.21	–	0.60	6.12	2*n* = 2*x*	S	1	1	1	IS	First estimation
*Linaria spartea*	Plantaginaceae	1.11 ± 0.05	4.1	0.55	4.05	2*n* = 2*x*	S	29	149	9	POP + IS	First estimation
*Linaria supina*	Plantaginaceae	1.30 ± 0.03	2.7	0.65	3.76	2*n* = 2*x*	S	9	57	3	POP	First estimation
*Linaria thriornithophora*	Plantaginaceae	2.66 ± 0.08	3.0	1.33	3.03	2*n* = 2*x*	S	14	98	5	POP	First estimation
*Melampyrum pratense* subsp*. latifolium*	Orobanchaceae	15.69 ± 0.19	1.2	7.84	3.27	2*n* = 2*x*	P	6	46	2	POP	First estimation
*Misopates calycinum*	Plantaginaceae	0.88 ± 0.04	4.4	0.44	4.04	2*n* = 2*x*	S	3	26	1	POP	First estimation
*Misopates orontium*	Plantaginaceae	0.88 ± 0.04	4.3	0.44	4.91	2*n* = 2*x*	S	19	97	7	POP	First estimation
*Northobartsia asperrima*	Orobanchaceae	1.55 ± 0.02	1.3	0.77	3.58	2*n* = 2*x*	S	3	27	1	POP	First estimation
*Odontite vernus*	Orobanchaceae	1.16 ± 0.02	1.8	0.58	4.05	2*n* = 2*x*	S	3	30	1	POP	First estimation
*Odontitella virgata*	Orobanchaceae	4.27 ± 0.02	0.5	2.13	2.93	2*n* = 2*x*	G/S	6	60	2	POP	First estimation
*Parentucellia viscosa*	Orobanchaceae	2.72 ± 0.06	2.0	1.36	2.83	2*n* = 2*x*	S	6	24	3	POP	First estimation
*Pedicularis sylvatica* subsp*. lusitanica*	Orobanchaceae	5.95 ± 0.15	2.5	2.97	2.42	2*n* = 2*x*	S	8	29	3	POP	First estimation
*Pedicularis sylvatica* subsp*. sylvatica*	Orobanchaceae	5.61 ± 0.02	0.3	2.81	3.15	2*n* = 2*x*	S	2	2	1	POP	First estimation
*Rhinanthus minor*	Orobanchaceae	2.81 ± 0.08	2.8	1.40	5.26	2*n* = 2*x*	P	3	20	1	POP	2C = 7.9 pg^B^
*Scrophularia auriculata* subsp. *auriculata*	Scrophulariaceae	1.79 ± 0.01	1.6	0.90	3.98	2*n* = 2*x*	P	8	8	1	IS	First estimation
*Scrophularia frutenscens*	Scrophulariaceae	1.34 ± 0.03	2.5	0.67	5.55	2*n* = 2*x*	P	7	34	3	POP + IS	First estimation
*Scrophularia grandiflora*	Scrophulariaceae	1.94 ± 0.07	6.6	0.97	4.13	2*n* = 2*x*	B/G/P	19	51	6	POP + IS	First estimation
*Scrophularia herminii*	Scrophulariaceae	2.56 ± 0.07	2.7	1.28	6.15	2*n* = 2*x*	P	3	16	1	IS	First estimation
*Scrophularia lyrata*	Scrophulariaceae	3.19 ± 0.05	0.7	1.60	4.54	2*n* = 2*x*	P	3	15	1	POP	First estimation
*Scrophularia nodosa*	Scrophulariaceae	1.19 ± 0.01	0.6	0.60	6.71	2*n* = 2*x*	P	2	2	1	IS	First estimation
*Scrophularia sambucifolia* subsp. *sambucifolia*	Scrophulariaceae	1.86 ± 0.04	2.0	0.93	4.33	2*n* = 2*x*	P	5	5	1	POP	First estimation
*Scrophularia scorodonia*	Scrophulariaceae	2.11 ± 0.05	2.2	1.06	4.57	2*n* = 2*x*	B/G/P	19	106	7	POP + IS	First estimation
*Scrophularia sublyrata*	Scrophulariaceae	2.22 ± 0.12	5.5	1.11	5.95	2*n* = 2*x*	B	5	15	2	POP	First estimation
*Verbascum levanticum*	Scrophulariaceae	0.75 ± 0.02	2.9	0.38	5.57	2*n* = 2*x*	R	3	16	1	POP	First estimation
*Verbascum litigiosum*	Scrophulariaceae	0.76 ± 0.03	4.2	0.38	3.48	2*n* = 2*x*	S	3	30	1	POP	First estimation
*Verbascum pulverulentum*	Scrophulariaceae	0.78 ± 0.02	2.2	0.39	4.15	2*n* = 2*x*	S	3	30	1	POP	First estimation
*Verbascum simplex*	Scrophulariaceae	0.74 ± 0.02	2.8	0.37	3.70	2*n* = 2*x*	S	12	70	4	POP	First estimation
*Verbascum sinuatum*	Scrophulariaceae	0.77 ± 0.04	4.7	0.39	5.08	2*n* = 2*x*	S	17	121	6	POP + IS	First estimation
***Verbascum virgatum***	Scrophulariaceae	1.44 ± 0.02	1.5	0.36	3.51	**2n = 4*x***	S	4	11	2	POP + IS	First estimation
*Veronica acinifolia*	Plantaginaceae	1.24 ± 0.01	0.7	0.62	3.73	2*n* = 2*x*	S	3	3	1	POP	First estimation
*Veronica arvensis*	Plantaginaceae	0.91 ± 0.01	1.6	0.46	3.92	2*n* = 2*x*	S/R	9	58	3	POP	2C = 0.66 pg^C^
***Veronica chamaedrys* subsp. *chamaedrys***	Plantaginaceae	3.72 ± 0.02	0.6	0.62	3.70	**2n = 6*x***	G/S	3	30	1	POP	2C = 2.98 pg^C, D^
***Veronica hederifolia***	Plantaginaceae	4.16 ± 0.08	2.0	0.69	2.84	**2n = 6*x***	B	3	8	1	POP	2C = 2.82 pg^C^
***Veronica micrantha***	Plantaginaceae	2.15 ± 0.04	1.7	0.54	7.56	**2n = 4*x***	P	3	17	1	IS	First estimation
***Veronica officinalis***	Plantaginaceae	2.10 ± 0.06	2.9	0.53	3.98	**2n = 4*x***	B/P	12	51	4	POP	First estimation
***Veronica peregrina* subsp. *peregrina***	Plantaginaceae	1.96 ± 0.06	2.9	0.49	4.02	**2n = 4*x***	B	3	8	1	POP	2C = 1.90 pg^C^
*Veronica persica*	Plantaginaceae	1.40 ± 0.03	2.4	0.35	4.70	2*n* = 4*x*	S	24	105	7	POP	2C = 1.55 pg^C, D^
*Veronica polita*	Plantaginaceae	0.77 ± 0.01	1.5	0.39	4.61	2*n* = 2*x*	S	6	18	2	POP	2C = 0.84 pg^C^

**Fig. 2:**
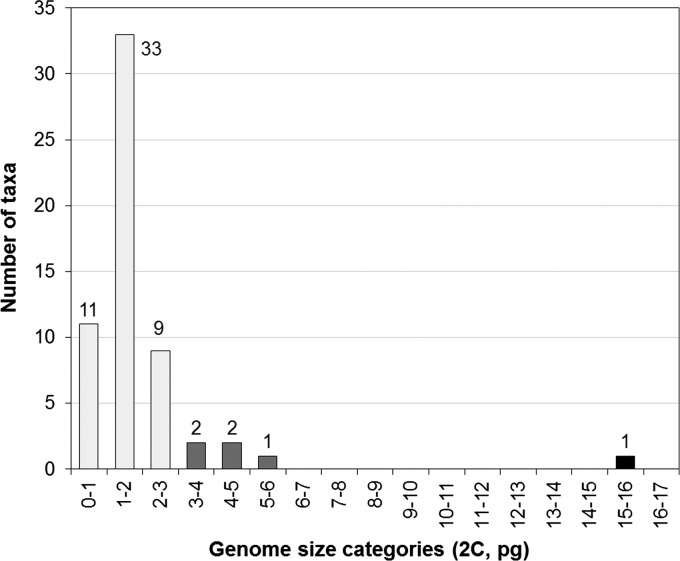
**Distribution of genome size according to genome categories (1 pg difference).** Colours represent the categories defined by Leitch *et al*. (1998).

Among the species sampled, a genome size variation of 21.6-fold was found, with the lowest mean value being obtained for *Verbascum simplex* (2C = 0.74 ± 0.02 pg) and the highest one for *Melampyrum pratense* subsp. *latifolium* (2C = 15.69 ± 0.19 pg). Still, according to the genome size categories defined by Leitch *et al*. (1998), 89.8 % of the taxa have a very small genome (2C ≤ 2.8 pg), 8.5 % have a small genome (2.8 pg < 2C ≤ 7.0 pg) and 1.7 % have an intermediate genome (7.0 pg < 2C ≤ 28.0 pg). No species with large (28.0 pg < 2C ≤ 70.0 pg) and very large (2C > 70.0 pg) genome sizes were detected (Fig. 2). No significant differences in genome size were obtained among families considering the newly established taxonomy (Kruskal–Wallis one-way ANOVA: *H*_2_ = 5.47, *P* = 0.065).

**Fig. 3:**
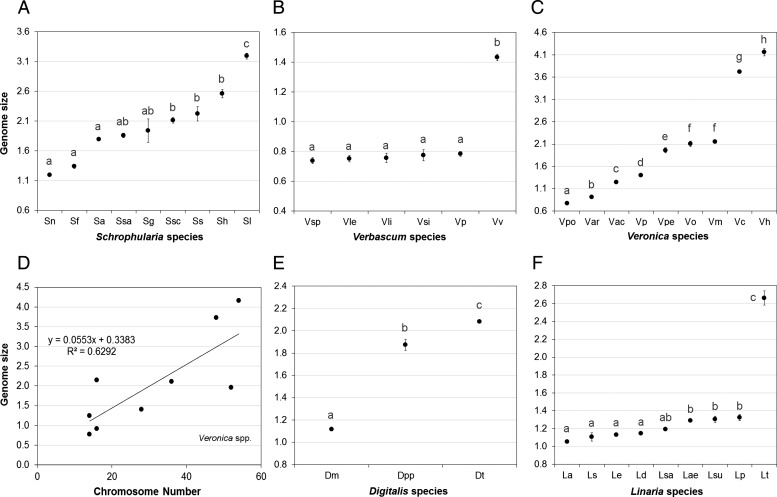
**Genome size variation (mean and standard deviation of the mean) in Scrophulariaceae genera.** (A) *Scrophularia* spp. (Sn, *S. nodosa*; Sf, *S. frutescens*; Sa, *S. auriculata* subsp. *auriculata*; Ssa, *S. sambucifolia* subsp. *sambucifolia*; Sg, *S. grandiflora*; Ssc, *S. scorodonia*; Ss*, S. sublyrata*; Sh*, S. herminii*; Sl, *S. lyrata*); (B) *Verbascum* spp. (Vsp, *V. simplex*; Vle, *V. levanticum*; Vli, *V. litigiosum*; Vsi, *V. sinuatum*; Vp, *V. pulverulentum*; Vv, *V. virgatum*); (C) *Veronica* spp. (Vpo, *V. polita*; Var, *V. arvensis*; Vac, *V. acinifolia*; Vp, *V. persica*; Vpe, *V. peregrina* subsp. *peregrina*; Vo, *V. officinalis*; Vm, *V. micrantha*; Vc, *V. chamaedrys* subsp.*chamaedrys*; Vh, *V. hederifolia*); (D) linear regression between mean nuclear DNA content and chromosome number of *Veronica* spp. (linear regression equation and *R*^2^ coefficient are also provided); (E) *Digitalis* spp. (Dm, *D. mariana*; Dpp, *D. purpurea* subsp. *purpurea*; Dt, *D. thapsi*); (F) *Linaria* spp. (La, *L. amethystea* subsp. *amethystea*; Ls, *L. spartea*; Li, *L. incarnata*; Ld, *L. diffusa*; Lsa, *L. saxatilis*; Lae, *L. aeruginea* subsp. *aeruginea*; Lsu, *L. supina*; Lp, *L. polygalifolia* subsp. *polygalifolia*; Lt, *L. triornithophora*). Different letters represent groups that are significantly different (*P* < 0.05).

A detailed analysis of the variation in genome size within each genus revealed that no statistically significant differences were detected in genome size among sampled taxa of *Anarrhinum* (ANOVA: *F*_2_ = 1.51, *P* = 0.230), *Anthirrhinum* (ANOVA: *F*_4_ = 2.39, *P* = 0.082) and *Misopates* (*t*-test: *t* = 0.01, *P* = 0.991). In all the other sampled genera, statistically significant differences were observed [see details of the tests in Additional Information; Fig. 3], with genome size being an important character to separate at least two taxa within each genus.

In *Scrophularia*, *Verbascum* and *Veronica* genome size differences were due to different numbers of chromosomes among taxa. Also, despite many *Scrophularia* species not being statistically different, due to dissimilar and non-overlapping values of genome size, it was possible to use this character to separate several species (e.g. *S. nodosa*, *S. frutescens*, *S. hederifolia*, *S. lyrata*; Kruskal–Wallis one-way ANOVA: *H*_8_ = 62.72, *P* < 0.001; Fig. 3A). In *Verbascum*, despite being possible to statistically distinguish *V. virgatum* from all the other analysed taxa, the remaining ones had very similar genome sizes (ANOVA: *F*_5_ = 374.31, *P* < 0.001; Fig. 3B). In *Veronica*, with the exception of *V. officinalis* and *V. micrantha*, all the other analysed taxa were significantly different in genome size (ANOVA: *F*_8_ = 1677.4, *P* < 0.001; Fig. 3C). The linear regression analysis between chromosome numbers and genome size revealed a positive correlation between these characters, with a relatively high *R*^2^ value of 0.7229 (Fig. 3D); a Pearson correlation analysis confirmed this result (correlation coefficient of 0.85, *P* *<* 0.05).

In the case of *Digitalis*, *Linaria* and *Pedicularis*, according to the literature, all the analysed taxa within each genus present the same number of chromosomes (56, 14 and 16, respectively [see Additional Information]). However, regardless of that, statistically significant differences in genome size were detected [see Additional Information], being possible to separate the analysed species of *Digitalis* (ANOVA: *F*_2_ = 129.93, *P* < 0.001; Fig. 3E) and *Pedicularis*. In *Linaria*, *L. triornithophora* presented a statistically distinguishable higher genome size than the remaining species (ANOVA, *F*_8_ = 750.99, *P* < 0.001; 2C = 2.66 pg; Fig. 3F); however, the other species presented dissimilar but close 2C values of genome size ranging from 1.05 to 1.32 pg, not all distinguishable statistically (Fig. 3F).

In taxa where more populations were collected, the incidence of intraspecific variation of genome size was evaluated. In *Digitalis purpurea* subsp. *purpurea* a DNA range of 1.76–2.06 pg/2C (*n* = 10 populations) was obtained [see Additional Information]. Still, in all populations except for population MC92 the estimates were homogeneous (CV < 2.0 %). In this population a CV value of 5.6 % was obtained, reflecting three dissimilar genome size estimates (1.80, 1.91 and 2.02 pg/2C). These results at population level reflected the scenario obtained for this taxon, with three main groups of estimates differing by approximately 0.11 pg being obtained between populations [see Additional Information]. In *Linaria* spp., usually low CV values of genome size (<3.0 %) were obtained among populations of the same taxa. Still, in *L. triornithophora* and *L. spartea* higher CV values were found [see Additional Information]. In *Scrophularia* and *Verbascum*, some heterogeneity in genome size values was found within some species (e.g. *S. grandiflora*), with differences both among and within populations being detected (data not shown). In *Verbascum*, some heterogeneity in genome size estimates was observed, with 7 out of 15 populations of different taxa presenting genome size CV values higher than 3.5 %, mostly due to within-population variability and/or instrument-related variability. In *Veronica*, all species and populations presented homogeneous genome size estimations.

Concerning the incidence of polyploidy in Scrophulariaceae, in contrast to what was expected, at least for some taxa, no different cytotypes were detected among any of the 162 surveyed populations in any of the 59 taxa. Nevertheless, as referred to above, within some genera (e.g. *Veronica*, *Verbascum*) there were species with different DNA ploidy levels (*sensu* Suda *et al*. 2006). In the particular case of *Veronica*, according to the 1Cx analysis presented in Fig. 3D, four novel DNA ploidy levels were assumed, namely 6*x* populations in *V. chamaedrys* subsp. *chamaedrys* and *V. hederifolia*, and 4*x* populations in *V. officinalis* and *V. micrantha* (Table 1).

## Discussion

The amount of DNA per chromosome set is known to be a fairly constant characteristic of a species. Therefore, during the past decade an increasing interest on genome size studies and its significance has been observed, with many studies focused on using genome size as a taxonomic marker and on finding correlations between ecological and environmental variables and this character. However, there are still many families being neglected, including Scrophulariaceae, for which the present study contributed more data than that available so far. Furthermore, due to the importance of polyploidy events in the genesis of new entities, it is important to evaluate how common these events are in nature. The detailed bibliographic analyses of polyploidy incidence in this family seemed to indicate that at least some taxa could present different cytotypes. However, the absence of more than one cytotype in all the analysed species revealed that polyploidy apparently is not among the main mechanisms of current speciation in Scrophulariaceae, at least in this region.

After molecular studies using DNA sequences of plastid genes, genera belonging to Scrophulariaceae *s.l.* were reorganized into six different families (Olmstead *et al*. 2001). A comparison of genome size taking into consideration this new classification did not reveal any pattern. This result was expected, as genome size estimations obtained in Scrophulariaceae *s.l.* fell almost exclusively in the very small and small genome size categories (Leitch *et al*. 1998), presenting a relatively low variation.

As already observed in many genera (e.g. *Helleborus*, Zonneveld *et al.* 2001) genome size can be used as an extra taxonomic character for discriminating between closely related taxa. Species belonging to *Bartsia*, *Nothobartsia* and *Parentucellia* share a close evolutionary history and some morphological similarities. This has been reflected in different generic circumscriptions, with *Nothobarsia asperrima* having been formerly included in the genus *Bartsia* as *Bartsia asperrima* (Benedí *et al*. 2009). The same situation is repeated with species belonging to *Odontites* and *Odontitella* (Benedí *et al*. 2009). All the analysed species, and thus genera in particular circumscriptions, had non-overlapping genome sizes, and thus in case any doubt should arise in species identification, using genome estimates the assignment to a taxonomic category would be straightforward. In a similar study, Loureiro and co-authors were able to distinguish two genera of Ulmaceae, *Ulmus* and *Celtis* (Loureiro *et al*. 2007*b*).

A survey of the Plant DNA C-values database (Bennett and Leitch 2010) revealed a high incidence of intra-generic variation in genome size in homoploid species. At least two-fold variation in monoploid genome size was recorded for more than one-third of the genera for which there was sufficient coverage of homoploid species (Suda *et al*. 2006). Genera where detailed studies on genome size variation were already performed include *Hydrangea* (Cerbah *et al.* 2001), *Artemisia* (Torrell and Vallès 2001), *Elytrigia* (Mahelka *et al*. 2005) and *Curcuma* (Leong-Škorničková *et al.* 2007), among others. In the case of Scrophulariaceae, contrasting results were obtained among the studied genera: while in a few (*Anarrhinum*, *Antirrhinum* and *Misopates*), genome size was an unsuitable character for taxonomic purposes, as all the estimates were very homogeneous among species, in the other analysed genera, genome size could be used for taxa delimitation and for analyses of interspecific variation, especially in the homoploid taxa *Digitalis*, *Pedicularis* and *Linaria*.

In the particular case of *Digitalis*, all the analysed species had different genome sizes, and these data support recent taxonomic changes in this genus: traditionally, *Digitalis mariana* was considered one sub-species of *Digitalis purpurea* and has recently been elevated to the species level (Benedí *et al*. 2009). Indeed, this new species presents a genome size significantly lower than that of *D. purpurea* subsp. *purpurea*. It will be very interesting to apply FCM to all the species in the genus and evaluate if it continues to be possible to discriminate these homoploid taxa using genome size.

In *Linaria*, with the exception of *L. triornitophora*, which presented a higher genome size value, all the other species presented more similar genome sizes; nevertheless, due to the high quality of the obtained estimates, it was possible to use this character to separate some taxa. However, two commonly confused taxa, *L. polygalifolia* subsp. *polygalifolia* and *L. supina*, presented the same genome size and thus, unfortunately, could not be distinguished using this character. A rough analysis considering the subgeneric level seems to indicate that members of section *Pelisserianae* present the highest values of genome size, while those from section *Versicolores* present the lowest. Still, this can be due to the reduced number of species analysed in those sections, as evident by the larger heterogeneity in genome size observed in section *Supinae*, the section to which most of the analysed species belong. Another approach could be to consider that the analysed individuals of *L. triornitophora* present double the number of chromosomes than all the other analysed taxa. Future studies using classical chromosome counts need to be done to confirm this possibility.

In *Veronica*, *Scrophularia* and *Verbascum*, most of the observed differences in genome size were related to different chromosome numbers. Still, considering that obtaining good microscopic plates for counting chromosome numbers in all the analysed species would take a long time, the value of genome size estimates is undeniable also in these cases. Using this character, it was possible to distinguish all the analysed taxa of *Veronica*, with the exception of *V. micrantha* and *V. officinalis*. In a comparison with the only genome size study focused on this genus, some of our estimates are very similar to those of Albach and Greilhuber (2004) (e.g. *V. peregrina* subsp. *peregrina*), while others are clearly different (e.g. *V. chamaedrys* subsp. *chamaedrys* with 3.72 pg/2C in this study vs. 2.98 pg/2C in the literature, and *V. arvensis* with 0.91 pg/2C in this study vs. 0.66 pg/2C in the literature). Some of these differences could easily be justified by different ploidy levels, as is possibly the case for *V. chamaedrys* and *V. hederifolia* where hexaploidy was assumed in our case instead of the reported tetraploidy (Albach and Greilhuber 2004). Still, in the case of *V. arvensis* the large difference that we observed may be related to the use of different techniques and methodologies. Indeed, most of the estimates reported by Albach and Greilhuber (2004) were obtained using Feulgen densitometry, including that of *V. arvensis*. Despite Doležel *et al*. (1998) having shown a close agreement between both methods, there are numerous cases in the literature where estimates obtained using both techniques do not correspond. For example, Loureiro *et al.* (2007*a*, *b*) using FCM obtained a 2C value of 5.08 pg DNA for *Coriandrum sativum*, while Das and Mallick (1989) using Feulgen microdensitometry obtained 2C values ranging between 7.65 and 9.55 pg/2C.

These differences may be related to the many critical points of the Feulgen technique (e.g. fixation, slide preparation and storage, acid hydrolysis), which are not always followed and that may influence the obtained estimations (Greilhuber 1988). Particularities of the FCM methodology, such as the use of different reference standards, sample preparation and staining protocols (Doležel *et al*. 1998), may also contribute to these differences. Also, following the linear regression between chromosome numbers and genome size, it seems that the analysed individuals of *V. micrantha* and *V. officinalis* are tetraploid and not diploid, as reported in the literature. Also, in the case of *V. officinalis*, there are some previous reports of 36 chromosomes with two base chromosome numbers, 9 and 18, being reported (Benedí *et al*. 2009), indicating some confusion as to what ploidy level the set of 36 chromosomes corresponds. However, as these are the first estimates of genome size, classic karyological analyses should be performed in the future to fully confirm these assumptions.

In *Scrophularia*, several species had apparently different genome sizes, but those differences were revealed to be not statistically significant (most likely due to the use of a non-parametric statistical test). In this genus, the species with the highest number of chromosomes, *S. auriculata* subsp. *auriculata*, is not the one with the larger value of genome size. Considering the number of chromosomes that this species presents (78–88 chromosomes) it is certain that several polyploidy events occurred in the past and, as happened in other species (e.g. *Nicotiana* spp., Leitch *et al*. 2008), these phenomena may have been accompanied by genome downsizing. It is assumed that DNA loss during polyploidization may be a selection mechanism to lessen genetic instability or the phenotypic effects of having a larger nucleus and cell size (Leitch *et al*. 2008).

Finally, in *Verbascum*, if we exclude the tetraploid *V. virgatum* with approximately double the value of genome size of the remaining species, the other taxa presented very similar genome sizes. Still, all these species present different chromosome numbers (i.e. 30, 32, 36 chromosomes; Benedí *et al*. 2009). This may be due to a phenomenon called dysploidy, i.e. the increase or decrease of one or a few chromosomes. The decrease in chromosome numbers appears not to be usual (Martel *et al*. 2004; Hidalgo *et al*. 2007) and is commonly attributed to the fusion of two or more chromosomes. In principle, this would not affect the genome size in any way. Based on the chromosome number variation, descendant dysploidy has been suggested for several genera of Iridaceae (Goldblatt and Takei 1997). For example, in *Iris* subgenus *Xiphium*, it was proposed that if the ancestral base number was *x* = 9, and *I. boissieri* (*n* = 18) represented a polyploidy event, descending dysploidy may explain the remaining chromosome numbers (*n* = 17, 16, 15, 14). In a similar way, in *Verbascum*, chromosome fusions may explain a decrease in the number of chromosomes from 36, to the remaining chromosome numbers that are reported in the literature, without variation in genome size. Molecular cytogenetic techniques such as fluorescence *in situ* hybridization could be used in the future to clarify this hypothesis.

The analysis of intraspecific variation revealed some variation in genome size among individuals of the same species, both among and within populations. While some authors argue for a large plasticity of the nuclear genome, others claim a more stable genome size within species. In recent years, several reports that followed best practices confirmed the existence of this phenomenon (see Šmarda and Bureš 2010 for a review). In the case where there is a true intraspecific variation, chromosomal differences (aneuploidy and supernumerary B-chromosomes) and polymorphisms in A-chromosomes (heterochromatic knobs and differential deletion of transposable element remnants; Gregory 2005*a*) may explain the differences that were reported. In particular, it is worth highlighting the differences observed in the genome size estimates among individuals of *D. purpurea* subsp. *purpurea*. In this subspecies, three groups differing by ∼0.11 pg were observed. Despite some geographical relationship being found among these three genome size groups, with the higher genome sizes being found when heading north of Portugal and Galicia, it is worth noticing that all three groups were detected in one of the populations (MC92). In the literature, two chromosome numbers, 48 and 56, are known (Benedí *et al*. 2009). Furthermore, the possibility of presenting B chromosomes is documented for this species (Regnart 1934). A joint effect of these events may contribute to the intraspecific variation observed in this subspecies, similar to what was reported by Sharbel *et al*. (2004) in *Boechera holboellii*.

## Conclusions and forward look

In conclusion, this work contributed important basic scientific knowledge on genome size and polyploid incidence in the Scrophulariaceae, providing important background information for subsequent studies, namely taxonomic studies in some interesting groups and focused on the ecological significance of genome size and polyploidy and their importance in plant diversification in this region. Indeed, regarding genome size evolution, several doors were opened, with intraspecific variation of genome size and dysploidy being among the most interesting detected phenomena to be explored in the future.

## Additional information

The following Additional Information is available in the online version of this article –

**File 1.** Table. Lists the plant material of Scrophulariaceae *s.l*. analysed in this study.

**File 2.** Table. Summarizes the bibliographic review on chromosome counts and distribution within the Iberian Peninsula.

**File 3.** Table. Lists the results of the statistical analyses performed in this study.

**File 4.** Table. Lists the genome size estimations in the taxa studied for *Digitalis*.

**File 5.** Table. Lists the genome size estimations in the taxa studied for *Linaria*.

**File 6.** Table. Lists the reference standards used in this study and their genome sizes.

**File 7.** Figure. Exemplifies how flow cytometric data were acquired.

## Sources of funding

The work was funded by the Portuguese Foundation for Science and Technology through the fellowship to S.C. (FCT/BPD/41200/2007).

## Contributions by the authors

J.L. and S.C. conceived the initial idea. M.C. performed the bibliographic review. M.C., J.L. and S.C. coordinated sampling and flow cytometric estimations. M.C. and J.L. analysed data and organized it in figures and tables. M.C. wrote the first draft of the manuscript. J.L. and S.C. edited the final version of the manuscript.

## Conflicts of interest statement

None declared.
